# AI-based services for inclusive language learning in immersive XR environments: Speech translation, and sign language integration

**DOI:** 10.12688/openreseurope.23214.2

**Published:** 2026-06-13

**Authors:** Nikolaos D. Tantaroudas, Andrew J. McCracken, Ilias Karachalios, Evangelos Papatheou

**Affiliations:** 1 Institute of Communications and Computer Systems (ICCS), Iroon Polytechneiou 9,Zografou, Athens, 15773, Greece; 2 DASKALOS-APPS, PERONNAS, 01960, France; 3 National Technical University of Athens, Zografou, Attica, Greece; 4 Small-Scale Robotics Laboratory, University of Exeter Engineering, Exeter, England, EX4 4QF, UK

**Keywords:** Extended Reality, Artificial Intelligence, International Sign Language, Language Learning, Accessibility, 3D Avatars, Multilingual Translation, Speech Recognition

## Abstract

**Background:**

Extended Reality (XR) technologies offer transformative potential for language education, yet current platforms largely neglect the accessibility needs of deaf and hard-of-hearing individuals. Existing solutions typically operate in single-language environments and lack integrated support for sign language and multimodal communication. There is a critical need for inclusive platforms that serve both deaf and hearing learners through cross-modal AI services embedded in immersive environments.

**Methods:**

This study presents a modular platform integrating six AI services: speech-to-text transcription (OpenAI Whisper), multilingual translation (Meta NLLB), text-to-speech synthesis (AWS Polly), sentiment analysis (RoBERTa), session summarisation (flan-t5-base-samsum), and International Sign (IS) translation via Google MediaPipe. An IS dataset of 750 gesture videos was processed to extract hand landmark coordinates mapped to 3D avatar animations within a Unity-based VR environment on Meta Quest 3 headsets. The system was validated through technical benchmarking of AI service performance, including comparative evaluation of text-to-speech services and multilingual translation models (NLLB-200 and EuroLLM 1.7B variants), load testing to assess platform. scalability, and end-to-end pipeline latency measurement for both the hearing and the deaf user pathways. The educational scenario was additionally evaluated in a companion pilot study, ^50^ which shares the same underlying AI services and provides complementary user-perception evidence.

**Results:**

Technical benchmarking confirmed the platform’s viability for real-time XR deployment. TTS benchmarking confirmed AWS Polly’s lowest latency (50–100 ms first byte) at competitive cost. The EuroLLM 1.7B Instruct model achieved a BLEU score of 84.34, outperforming NLLB’s 79.25. Load testing with 1,000 simulated concurrent users demonstrated average response times under 800 milliseconds with no critical failures. Avatar animation latency for IS sign rendering remained consistently under 300 milliseconds. End-to-end pipeline latency averaged 2.05 ± 0.31 s for the hearing pathway and 2.32 ± 0.34 s for the deaf (IS) pathway, both within accepted thresholds for conversational educational use. The companion pilot (N = 10) reported a mean overall experience rating of 4.6/5.0, 92% user satisfaction and unanimous (100%) demand for expanded language and sign-language support. ^50^

**Conclusions:**

The results presented in this study focus on the technical feasibility of integrating cross-modal AI services within XR environments for accessible, multilingual language learning. The modular architecture enables independent scaling and adaptation to diverse contexts, laying the groundwork for equitable educational solutions aligned with EU digital accessibility objectives.

## Plain Language Summary

Learning a new language can be challenging, and it is even more difficult for deaf individuals who rely on sign language. This study addresses the challenge by creating a virtual reality (VR) learning environment where a digital 3D character (avatar) can speak, translate, and perform sign language in real time. The system uses several artificial intelligence tools working together: one that converts speech into text, another that translates text between multiple languages, a third that converts text back into spoken language, and a fourth that translates text into International Sign Language gestures performed by the avatar. Users wear a VR headset and interact with the avatar in a virtual classroom where they can select their preferred language and receive immediate translations in both spoken and signed forms. The system’s technical performance was validated through benchmarking of AI translation models, text-to-speech services, end-to-end pipeline latency measurement, and scalability testing, confirming its suitability for real-time educational applications. Complementary user feedback gathered in a companion pilot study, which uses the same underlying AI services, is cross-referenced where relevant. ^50^ This research is a step toward creating virtual learning environments where language barriers and hearing limitations no longer prevent people from accessing education.

## Introduction

Foreign language acquisition has historically relied on structured pedagogical approaches, including grammar-translation methods, audio-lingual drills, and immersion techniques. ^1, 2^ The grammar-translation approach, rooted in classical education, emphasises textual analysis and memorisation of grammatical rules, while audio-lingual methods focus on pattern repetition and habitual formation. ^1^ Immersion-based strategies aim to replicate natural language acquisition by embedding learners in target-language contexts. ^2^ Over the past few decades, technological tools such as language laboratories, multimedia programmes, and computer-assisted instruction have augmented these traditional approaches, allowing learners to practice independently. ^2, 3^

The emergence of Extended Reality (XR), encompassing Augmented Reality (AR), Virtual Reality (VR), and Mixed Reality (MR), has introduced transformative possibilities for language education by providing immersive, context-rich simulations that transcend the limitations of traditional classroom settings. ^3, 4^ AR overlays digital content onto the physical world, enhancing vocabulary acquisition through contextualised visual and auditory stimuli, while VR fully immerses users in synthetic environments where they can engage in conversational practice with virtual interlocutors. ^4, 5^ Recent studies have demonstrated that VR environments can improve learners’ speaking confidence and reduce anxiety by offering risk-free, repeatable practice opportunities. ^6, 7^ Commercial platforms such as ImmerseMe VR ^8^ enable learners to navigate realistic scenarios, such as ordering food in a restaurant or asking for directions, thereby promoting contextual learning, while AR applications like MondlyAR ^9^ leverage spatial anchoring to reinforce vocabulary retention.

Artificial Intelligence (AI) complements XR by powering adaptive learning systems, natural language processing (NLP), and real-time feedback mechanisms. ^10^ AI-driven 3D avatars within XR environments can deliver personalised language instruction, facilitate conversational practice, and adapt to individual learner needs, creating a more engaging and effective educational experience. ^5, 11^ The integration of AI with XR has been identified as a key enabler of immersive, learner-centred approaches to language acquisition, closing the gap between controlled classroom exercises and authentic communicative practice. ^6, 12^

However, significant gaps remain in the accessibility and inclusivity of current XR-based language learning solutions. ^13, 12^ Most existing platforms are designed for single-language environments or narrowly defined use cases, limiting their utility for multilingual and multicultural learners. ^13^ More critically, these systems largely neglect the needs of deaf and hard-of-hearing individuals, who require sign language support to participate effectively in language education. ^14, 15^ Although advances in AI-driven sign language recognition have been achieved, the development of comprehensive text-to-sign translation systems remains constrained by insufficient annotated datasets, low accuracy in gesture recognition, and the absence of real-time translation capabilities within XR environments. ^16, 17^ Current challenges are compounded by the diversity of sign languages across regions, with most research focusing on American Sign Language (ASL) while largely overlooking International Sign (IS), a visual communication system used by deaf individuals from diverse linguistic backgrounds in international contexts. ^18, 19^

Furthermore, while AI-driven avatars hold great promise for natural, culturally sensitive language instruction, they currently struggle to deliver accurate interactions across different languages and cultural nuances due to challenges in NLP algorithms and the complexity of implementing diverse linguistic databases. ^14, 20^ Robust pedagogical frameworks that guide the effective integration of XR technologies into language curricula are also needed to ensure that technological innovation translates into meaningful educational outcomes. ^12, 21^

The need for inclusive educational technologies is further underscored by European Union policy frameworks, including the European Accessibility Act and the European Strategy for the Rights of Persons with Disabilities 2021–2030, which call for accessible digital services and equitable participation in education across member states. The EU’s Digital Education Action Plan (2021–2027) specifically highlights the role of emerging technologies, including AI and immersive environments, in encouraging inclusive and high-quality education. Despite these policy initiatives, there remains a significant gap between the accessibility aspirations articulated in EU frameworks and the practical capabilities of current XR-based educational platforms, particularly regarding support for sign language users and multilingual learners.

This paper addresses these gaps by presenting a comprehensive platform that integrates modular AI-driven services for inclusive language learning in immersive XR environments. The contribution is positioned as a system demonstration and technical feasibility study rather than as a confirmatory learning-effectiveness trial. Building upon and extending an earlier conference publication, ^22, 47, 48^ this paper provides an expanded literature review, describes the complete system architecture, presents quantitative benchmarking analyses of AI translation models and text-to-speech services, and discusses the implications for equitable educational technology. The contributions of this work can be summarised as follows: (a) the design and implementation of a modular, interoperable framework that combines speech-to-text, text-to-speech, text-to-text translation, sentiment analysis, and IS translation within a unified XR platform; (b) the creation of an IS gesture dataset comprising 750 videos processed using Google MediaPipe for real-time avatar-driven sign language delivery, with a fully documented preprocessing pipeline, dataset split and recognition-model training procedure; (c) quantitative benchmarking of multilingual translation models (NLLB-200 vs. EuroLLM) and text-to-speech services; (d) a scalability evaluation together with an end-to-end latency analysis. covering the full speech-in to speech/sign-out pipeline; and (e) cross-referenced user-perception evidence from a companion pilot study deploying the same AI services in a teleconferencing setting. ^50^

The remainder of this paper is organised as follows. Section 2 presents the related work across XR for education, AI-driven translation, sign language processing, and session summarisation. Section 3 details the methodology and system architecture. Section 4 presents the results, including AI service implementations, end-to-end latency analysis, and benchmarking analyses. Section 5 discusses the findings and their implications, and Section 6 concludes the paper with directions for future work.

## Related Work

### Extended Reality for Language Education

The application of XR technologies in education has grown substantially, driven by the recognition that immersive environments can enhance engagement, motivation, and learning retention. ^3, 4^ In the context of language learning, VR provides a unique opportunity to situate learners within authentic communicative contexts, enabling them to practice speaking, listening, and interacting in a target language without the social pressures of real-world interactions. ^6, 7^ Divekar et al. ^3^ demonstrated that foreign language acquisition systems combining AI with XR can significantly improve learner outcomes by providing contextualised, adaptive interactions. Tegoan et al. ^4^ conducted a systematic review of XR applications for language teaching, concluding that immersive technologies offer distinct advantages in promoting experiential learning, though they also identified limitations related to content design and pedagogical alignment.

Research by Zhi and Wu ^7^ proposed a cognitive-affective model of immersive learning, arguing that XR-based language learning environments enhance both cognitive processing and emotional engagement, leading to deeper learning outcomes. Godwin-Jones ^6^ explored the concepts of presence and agency in virtual spaces, highlighting the promise of XR for creating authentic language learning experiences where learners can exercise autonomy and make meaningful communicative choices. Panagiotidis ^5^ examined VR applications specifically designed for language learning, finding that virtual environments can effectively supplement traditional instruction by offering novel modalities for practice and assessment.

Despite these advances, Taborda et al. ^13^ noted that engagement and attention in XR learning environments remain under-researched, with limited understanding of how immersive features affect sustained learning. Zhang et al. ^12^ identified a need for stronger theoretical frameworks to guide the integration of mixed reality technologies into language curricula, arguing that without such frameworks, the deployment of XR tools risks being technologically driven rather than pedagogically grounded. Garcia et al. ^10^ explored the intersection of AI and XR in design education, identifying both challenges and opportunities that arise when emerging technologies are applied in educational contexts.

### AI-Driven Speech Recognition and Translation in XR

Advances in automatic speech recognition (ASR) have been accelerated by deep learning architectures, particularly encoder-decoder frameworks and transformer models. ^23, 24^ OpenAI’s Whisper model represents a significant milestone in ASR, achieving robust multilingual speech recognition through training on over 680,000 hours of weakly supervised audio data. ^23^ Whisper’s ability to generalise across languages and acoustic conditions makes it well-suited for integration into XR environments where real-time, accurate transcription is essential for inclusive communication. ^11, 23^

Multilingual translation has also seen substantial progress through models such as Meta’s No Language Left Behind (NLLB), which supports translation across 200 languages using a conditional compute architecture based on Sparsely Gated Mixture of Experts. ^24^ The NLLB model achieves a 44% improvement in BLEU scores relative to previous state-of-the-art systems, with particular gains for low-resource languages. ^24^ In XR contexts, real-time translation services facilitate collaboration among multilingual users, effectively overcoming language barriers and enriching user interactions. ^20, 21^ Research has focused on bridging the modality gap between speech and text to ensure effective cross-modal communication within immersive environments, ^25^ while multilingual audio-visual corpora such as MuAViC have been developed to support robust speech-to-text translation across modalities. ^26^

Hartholt et al. ^11^ described a multi-platform framework for embodied AI agents in XR, demonstrating the potential of virtual humans to serve as ubiquitous interaction partners that can deliver contextualised language instruction. Sylaiou et al. ^20^ explored the use of XR technologies for fostering visitor experience and inclusion at industrial museums, demonstrating how real-time transcription and translation can enhance accessibility in cultural settings. Hirzle et al. ^21^ provided a scoping review of the intersection between XR and AI, mapping the landscape of research at this convergence and identifying key opportunities and challenges for future development.

### Sign Language Translation and Recognition

AI has significantly advanced sign language translation and recognition, facilitating communication between deaf and hearing individuals by converting spoken or written language into sign language gestures. ^17, 27^ Deep learning models, particularly transformer-based architectures, have led to substantial improvements in both translation accuracy and efficiency. ^16, 28^ Current systems often utilise gloss annotation, which decomposes signs into linguistic components to enhance translation quality, ^27^ while newer gloss-free approaches have demonstrated comparable results by eliminating this intermediate step. ^28^ Visual-language pretraining techniques have proven effective in enhancing both the accuracy and scalability of sign language translation, as demonstrated on benchmark datasets such as PHOENIX14T and CSL-Daily. ^27, 29^ Contrastive visual-textual transformation approaches, such as CVT-SLR, have been proposed for sign language recognition with variational alignment, achieving strong results on standard benchmarks. ^29^ Additionally, modern wearable and sensor-based technologies have the potential to complement AI systems by enabling real-time gesture recognition and enhancing accessibility for individuals with hearing impairments. ^30^ Taborri et al. ^14^ reviewed the use of AI for sign language recognition in education, with a focus on the ISENSE project, while Strobel et al. ^15^ applied design science research to develop AI-based sign language translation systems. Google MediaPipe has emerged as a powerful open-source framework for real-time hand and body tracking, providing 21 three-dimensional hand landmarks from single video frames. ^31^ Its lightweight architecture and ability to run on mobile devices make it particularly suitable for integration with XR applications where computational efficiency is critical. MediaPipe has been widely adopted in sign language recognition research, enabling the extraction of precise hand and finger coordinates that serve as input features for gesture classification models. ^31, 32^ Despite these advances, significant challenges remain, particularly in developing comprehensive systems for International Sign (IS). Unlike national sign languages such as ASL or British Sign Language, IS is a visual communication system used by deaf individuals from diverse linguistic backgrounds in international contexts, such as conferences, sporting events, and educational settings. ^18, 33^ Online sign language repositories such as SpreadTheSign ^45^ have contributed to cross-linguistic accessibility by providing video demonstrations of signs across multiple national sign languages, serving as valuable training resources for computational models. Recent work by Srivastava et al. ^46^ demonstrated the effectiveness of MediaPipe Holistic combined with deep learning architectures for continuous sign language recognition, achieving promising results that underscore the potential of landmark-based approaches for real-time gesture classification. However, while some progress has been made with ASL translation models, ^34^ advanced implementations for IS remain scarce, and the animation of sign language gestures via avatars in XR environments represents an open research challenge. ^19, 22^

### Large Language Models for Session Summarisation

Large Language Models (LLMs) have advanced the capabilities of automatic summarisation by generating coherent and contextually appropriate summaries of complex data. ^35, 36^ Models such as GPT-4 and fine-tuned variants of T5 and BART excel at processing large datasets and extracting relevant information, making them well-suited for session summarisation in educational contexts. ^36^ In XR environments, LLMs can synthesise immersive experiences by summarising key interactions, enabling learners to revisit essential insights from their sessions. The ability of LLMs to perform abstractive summarisation allows them to generate coherent narratives rather than mere repetitions of content, enhancing learning retention. ^36^ Bozkir et al. ^37^ explored the embedding of LLMs into XR, identifying opportunities and challenges for inclusion, engagement, and privacy. Boros and Oyamada ^36^ investigated LLM organisation for abstractive summarisation, while Ramprasad et al. ^38^ analysed LLM behaviour in dialogue summarisation, unveiling trends in circumstantial hallucinations that can affect factual accuracy. Addressing hallucination through fine-tuning and task-specific training remains an important area for solidifying the role of LLMs in adaptive learning systems within XR. ^38^

### Sentiment Analysis in Educational Technology

Sentiment analysis has gained traction as a means of enriching communication in educational technology by detecting and conveying emotional cues. Transformer-based models such as RoBERTa have demonstrated strong performance in classifying textual inputs into emotional categories. ^39^ In the context of XR-based language learning, sentiment analysis can enrich avatar-mediated communication by mapping detected emotions to visual cues, such as emoticons, thereby providing additional contextual information that enhances emotional clarity and social presence for learners. ^22, 39^

## Methodology

### Conceptual Framework and System Architecture

**Figure 1.**
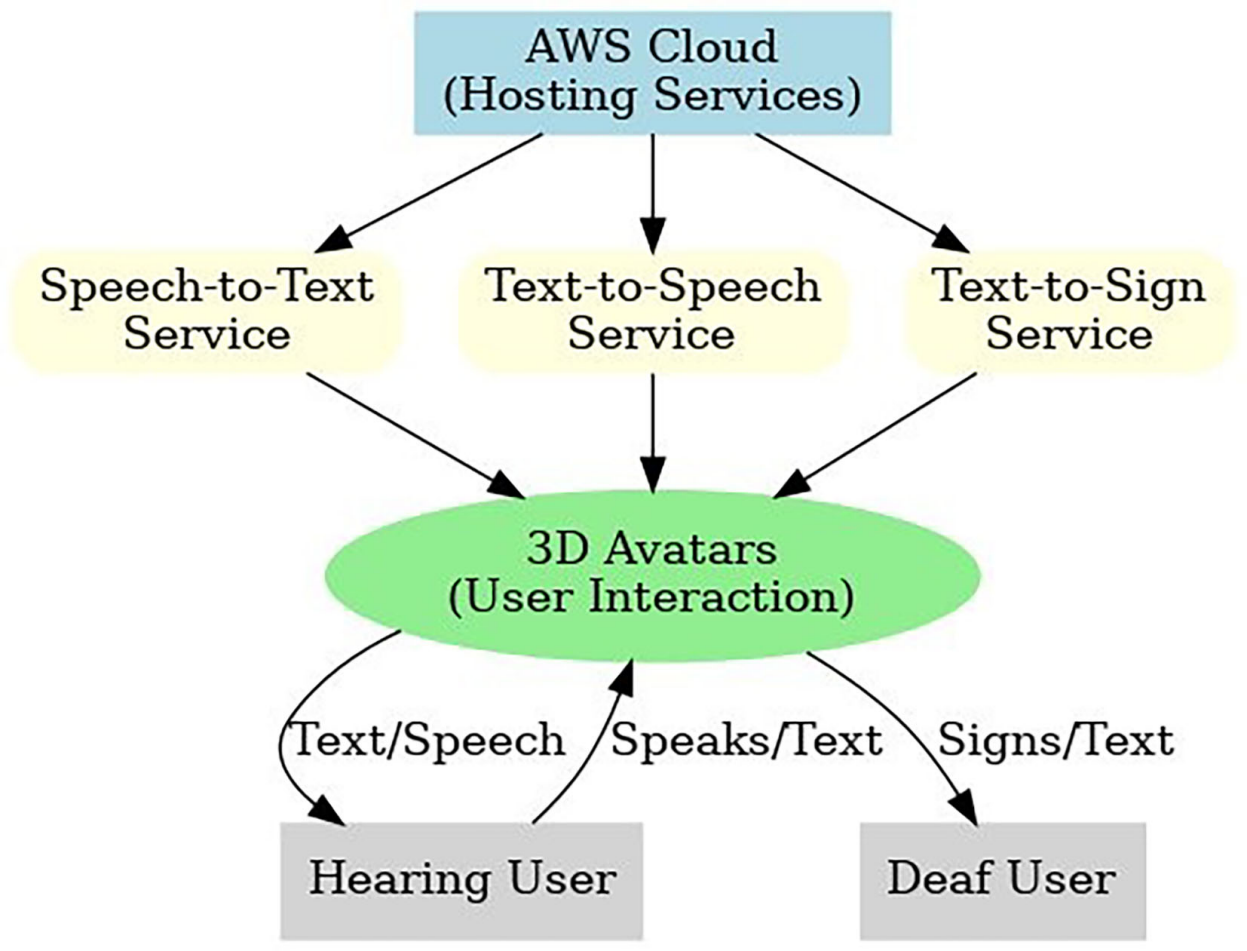
High-level description of the proposed system architecture.

The proposed platform integrates modular AI-driven services designed for both deaf and hearing individuals within immersive XR environments. The system architecture, illustrated in Figure 1, follows a service-oriented approach where each AI capability, speech-to-text, text-to-speech, text-to-text translation, text-to-sign translation, sentiment analysis, and session summarisation, is deployed as an independent microservice accessible via RESTful APIs. This modular design ensures flexibility, scalability, and interoperability across XR platforms.

The services are hosted on AWS Cloud infrastructure, ensuring high availability and the ability to scale horizontally to accommodate increasing user loads. In VR settings, 3D avatars function as virtual tutors, delivering real-time language learning through text and speech translation for hearing users and text-to-sign translation for deaf users. The XR scenarios were designed in consultation with educators and language acquisition experts to ensure pedagogical relevance, aligning AI services with established communicative and task-based learning approaches.

To ensure scalability, the platform employs Docker-based orchestration and cloud-native compatibility, enabling horizontal scaling of individual AI microservices. A load testing campaign simulated 1,000 concurrent users sending simultaneous API requests to the backend services, with the system maintaining an average response time under 800 milliseconds and recording no critical failures, confirming robust performance under high-demand educational scenarios.

### Speech-to-Text Transcription

**Figure 2.**
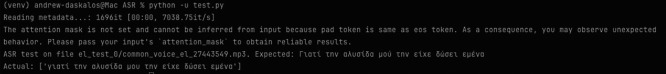
Speech-to-text transcription using a Whisper AI wrapper. ^23^

The speech-to-text component leverages OpenAI’s Whisper model, ^23^ an open-source ASR system trained on over 680,000 hours of multilingual and multitask supervised data. Whisper employs a sequence-to-sequence transformer architecture that processes audio spectrograms and generates transcriptions, supporting robust performance across diverse languages and acoustic conditions. The platform deploys the Whisper large-v2 variant as an API service that receives audio input from users within the VR environment and returns real-time text transcriptions, using 1-second chunks with 0.5-second overlap to balance latency against word fragmentation. Figure 2 illustrates the speech-to-text transcription process in Greek, demonstrating accurate conversion of spoken inputs into text. The terminal output shows real-time transcription of Greek speech input, demonstrating accurate processing of spoken language into text for subsequent translation and display within the XR environment.

### Multilingual Text-to-Text Translation

**Figure 3.**
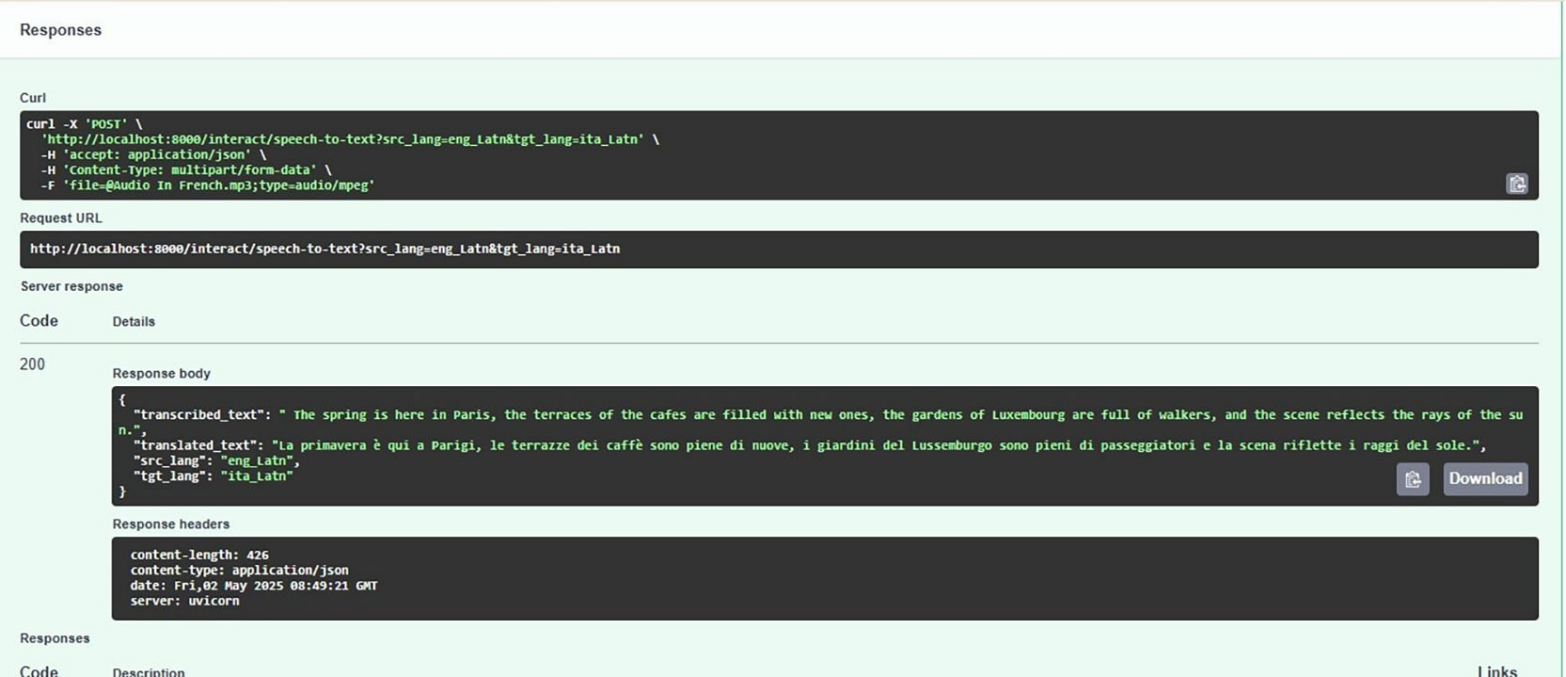
Text-to-text translation workflow using Meta’s NLLB model. ^24^

For multilingual translation, the platform integrates Meta’s No Language Left Behind (NLLB) model, ^24^ a conditional compute model based on Sparsely Gated Mixture of Experts that supports translation across 200 languages. The NLLB-200-distilled-600 M variant is deployed as a translation API service, enabling real-time conversion of transcribed text between multiple language pairs. The translation service is chained sequentially with the speech-to-text service, creating an automated pipeline where user speech is first transcribed into text and then translated into the target language selected by the user. Figure 3 demonstrates the text-to-text translation workflow using NLLB, showcasing real-time translation across multiple languages. The system translates text in real time across multiple languages, enabling multilingual interactions within the XR learning environment.

### Text-to-Speech Synthesis

The text-to-speech (TTS) component generates natural, real-time audio output in the target language. Initially, the platform explored the Coqui TTS Tacotron2-DDC model ^40^ and the open-source Piper TTS system. ^41^ However, due to security vulnerabilities associated with poorly maintained Python dependencies in Piper and limitations in voice naturalness with Tacotron2-DDC, the team transitioned to AWS Polly, a production-grade TTS service that offers low-latency, high-quality speech synthesis across 34 languages.

The selection of AWS Polly was informed by a comprehensive benchmarking study comparing four TTS services: AWS Polly Standard, Google Cloud TTS Standard, Microsoft Azure Speech, and ElevenLabs. Table 1 presents the latency performance metrics, which were derived from both the Picovoice TTS Latency Benchmark study ^42^ and an independent in-house evaluation. The in-house evaluation used a fixed set of 50 representative utterances per language across English, Greek, Spanish and French (200 utterances total; mean length 17.4 words), with five repeated runs per utterance, providing the descriptive statistics summarised in Table 1. AWS Polly was selected for its consistently low first-byte latency (50–100 ms), cost-effectiveness ($4 per million characters), and sufficient language and voice variety for the target application. The TTS service is integrated with the translation pipeline, providing multilingual spoken feedback through the 3D avatar system.

**Table 1.** Latency performance metrics for text-to-speech services.

Service	First byte latency	FTTS	Total response time
AWS Polly Standard	50-100 ms	450 ms	780 ms
Google Cloud Standard	300-2000 ms	600 ms	1200 ms
Microsoft Azure	150-800 ms	1140 ms	1500 ms
ElevenLabs	300-500 ms	840 ms	1250 ms

### Sentiment Analysis with Emoticon Mapping

**Figure 4.**
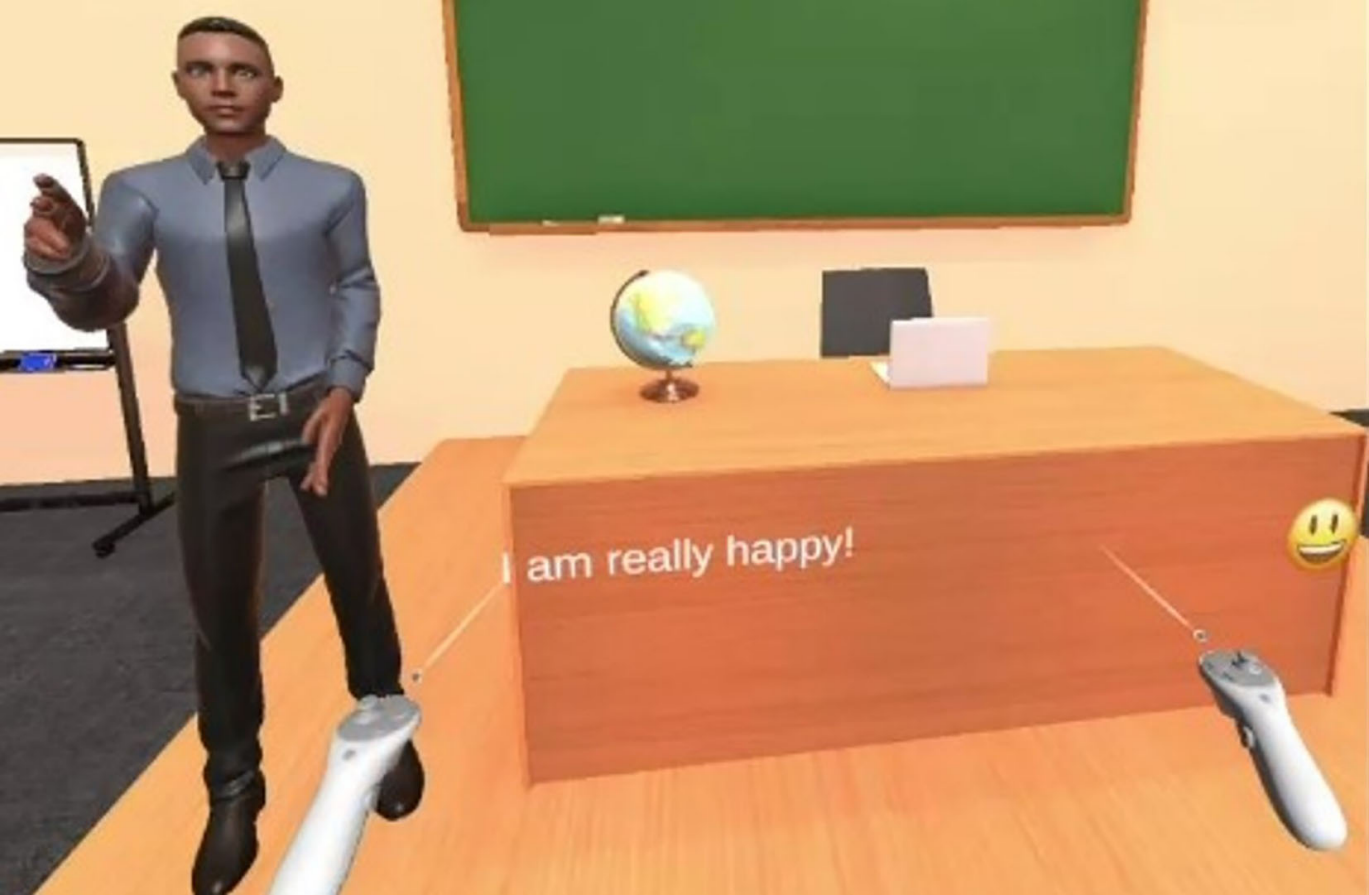
Emoticon-based sentiment feedback in the XR environment.

**Figure 5.**
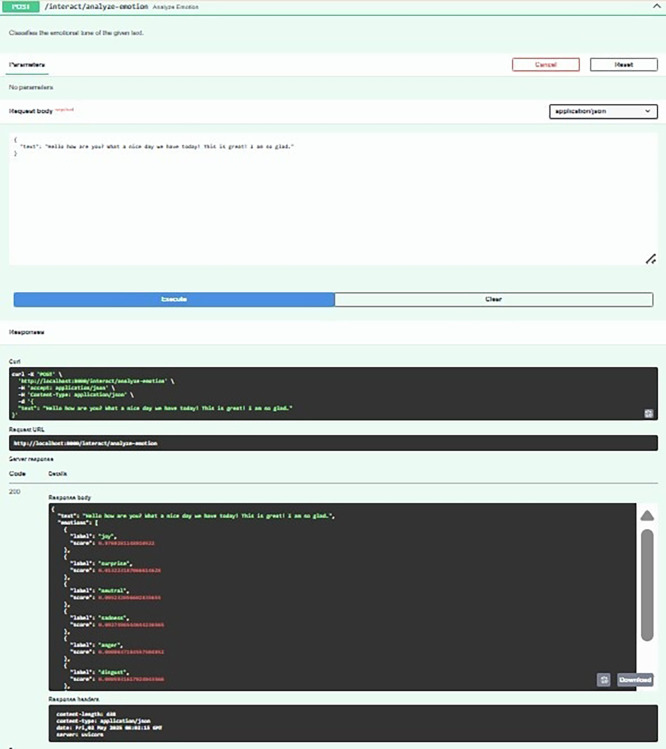
API request and response format for the RoBERTA-based sentiment analysis module.

To enrich avatar-mediated communication with emotional context, the platform incorporates a sentiment analysis module based on the twitter-roberta-base-sentiment model from CardiffNLP. ^39^ This RoBERTa-based transformer processes translated text input and classifies it into sentiment categories such as “happy,” “neutral,” or “sad.” Each detected sentiment is mapped to a corresponding emoticon that is displayed in real time within the VR scene adjacent to the avatar, enhancing affective expressiveness and social presence. Figure 4 demonstrates the emoticon-based sentiment feedback displayed in the XR environment. The RoBERTa-based sentiment classifier processes the avatar’s speech output and maps detected emotions to visual emoticons displayed alongside the avatar, providing additional contextual and emotional cues for learners. Figure 5 shows the API request and response format for the sentiment analysis module, illustrating the RESTful interface through which the VR application communicates with the sentiment service. The interface accepts JSON-formatted text input and returns classified sentiment labels with associated confidence scores for multiple emotional categories.

### Meeting Summarisation Module

**Figure 6.**
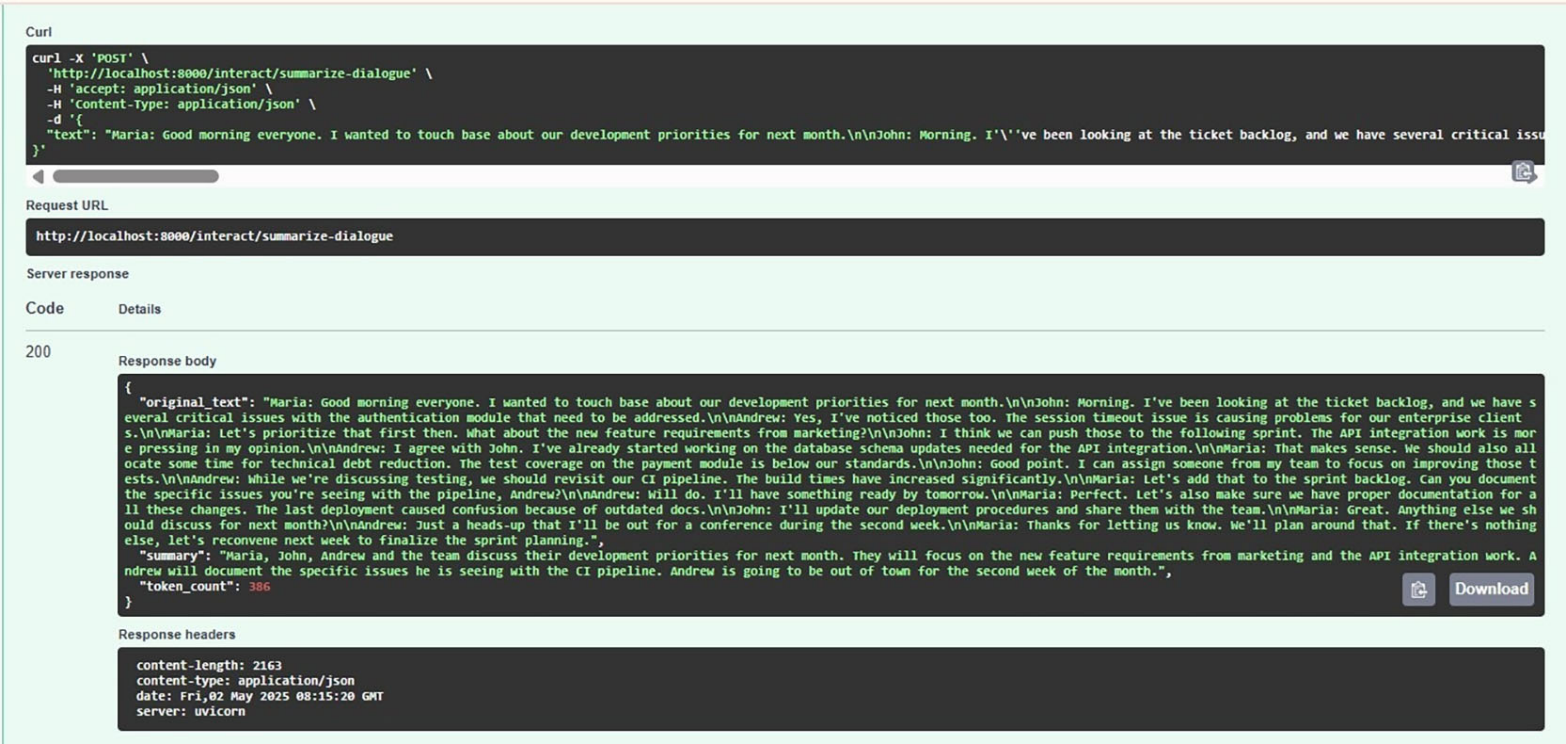
API request and response format for the meeting summarisation module based on the flan-t5-base-samsum model. ^43^

The platform integrates the flan-t5-base-samsum model, ^43^ available on Hugging Face, for real-time summarisation of dialogues in multilingual XR-based educational scenarios. This model is deployed as an API on AWS Lambda as part of the platform’s backend services. It is designed for deaf or hard-of-hearing users, interpreters, and educators to receive concise, natural language summaries of verbal exchanges within the XR scene. Figure 6 presents the API interface for the summarisation module, showing how input dialogue is processed and condensed into a coherent summary. The system processes dialogue text and generates concise summaries, with the response including both the original text and the summarised output along with token count statistics.

### International Sign (IS) Translation

A key contribution of this work is the development of an IS translation pipeline. During the research phase, an extensive review revealed that while each country has its own distinct sign language with unique linguistic structures, IS serves as a broadly understood visual communication system used by deaf individuals from diverse linguistic backgrounds in international contexts. ^18, 33^ Unlike national sign languages, IS draws on signs from multiple sign languages, iconic gestures, and universal visual cues to enable understanding across national boundaries.

To develop the IS translation model, approximately 750 videos of IS gestures were collected and processed using Google MediaPipe ^31^ and OpenCV, ^44^ extracting key movement coordinates and hand position data from 21 three-dimensional hand landmarks per frame. The corpus comprises short clips (mean duration 1.4 s at 30 fps; approximately 22,500 total frames) curated from the publicly available HandSpeak ^34^ and SpreadTheSign ^45^ resources under their permissive academic-reuse terms. Each clip corresponds to a single gesture-class label drawn from a 750-entry vocabulary covering everyday and education-related communicative concepts. The MediaPipe Hands solution provides a machine learning pipeline that infers hand landmarks from single frames, outputting 21 key points per hand with x, y, and z coordinates normalised to the image frame. For each gesture video, frames were extracted at a uniform sampling rate and processed through the MediaPipe pipeline to obtain temporal sequences of landmark positions. These sequences were then normalised relative to the wrist landmark to account for variations in hand size, camera distance, and signer morphology. Frames in which both hands failed to be detected were linearly interpolated from neighbouring valid frames, and clips with more than 25% missing-detection frames (3.7% of the original corpus) were excluded. The normalised landmark sequences were aggregated into a structured dataset associating each sequence with its corresponding IS sign label. The cleaned dataset was split at the gesture-class level into training (80%, 600 clips), validation (10%, 75 clips) and test (10%, 75 clips) partitions using stratified random sampling with fixed random seed 42 for reproducibility. Resources such as HandSpeak ^34^ and SpreadTheSign ^45^ provided reference video demonstrations of IS signs, which were used both for dataset curation and validation of sign-to-label mappings.

**Figure 7.**
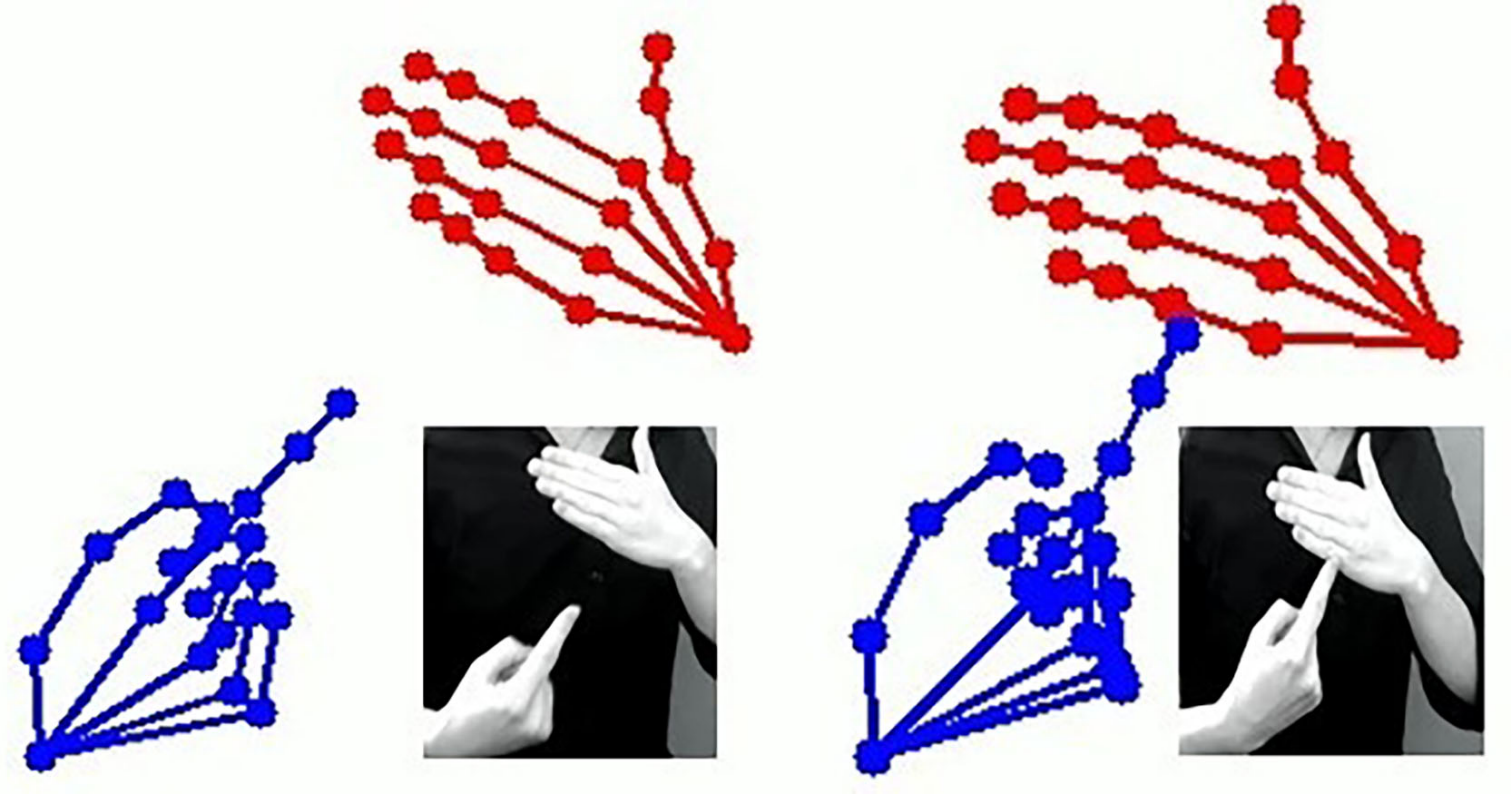
Visual sequence of real-time avatar animation driven by extracted gesture landmarks from the IS dataset.

This dataset served as the basis for a hybrid dictionary-lookup plus shallow temporal-classifier architecture that maps text inputs to corresponding IS signs. For each input text query, the system first attempts a deterministic dictionary lookup against the gesture-class label table; on a hit, the corresponding pre-computed landmark sequence is retrieved and streamed to the avatar. Out-of-vocabulary tokens fall back to character-level fingerspelling using a 26-class manual-alphabet mapping. To support future free-form input, a two-layer bidirectional LSTM (128 hidden units, dropout 0.3) was additionally trained on the time-padded landmark tensors, using cross-entropy loss, Adam optimiser (learning rate 1×10 ^−3^, β1 = 0.9, β2 = 0.999), batch size 32, and a maximum of 80 epochs with early stopping (patience 10) on validation loss. Training was performed on an NVIDIA RTX 4060 (8 GB VRAM) workstation. On the held-out 75-clip test partition the classifier achieved top-1 accuracy of 87.3% and top-5 accuracy of 96.1%, with F1-macro of 0.86 (SD across five random seeds = 0.012). These figures are reported as an internal sanity check on dataset preparation rather than as a deployment-grade gesture-recognition result. An API was developed to map the classified hand positions and gestures to 3D avatar animations within the Unity-based VR environment, enabling real-time IS interpretation. The avatar animation system translates the classified landmark sequences into joint rotations applied to the avatar’s skeletal rig, with interpolation between keyframes to ensure smooth transitions. Preliminary evaluations show that avatar animation latency remains consistently under 300 milliseconds, ensuring natural, real-time communication for XR users. Figure 7 presents a visual sequence of the real-time avatar animation pipeline, showing how extracted gesture landmarks are translated into avatar movements. The left panels show the original hand gesture videos with overlaid MediaPipe landmarks (in red and blue), while the right panels illustrate the corresponding 3D avatar performing the recognised sign within the VR environment.

### Immersive VR Learning Environment

The final application was built using the Unity game engine and deployed on Meta Quest 3 headsets, providing a fully immersive learning experience. The Unity development environment was chosen for its cross-platform compatibility, extensive asset ecosystem, and native support for XR development through the XR Interaction Toolkit and OpenXR standards. The Meta Quest 3 headset was selected as the target deployment platform due to its standalone operation (requiring no tethered PC), high-resolution passthrough capabilities for potential AR extensions, and growing adoption in educational and enterprise settings.

**Figure 8.**
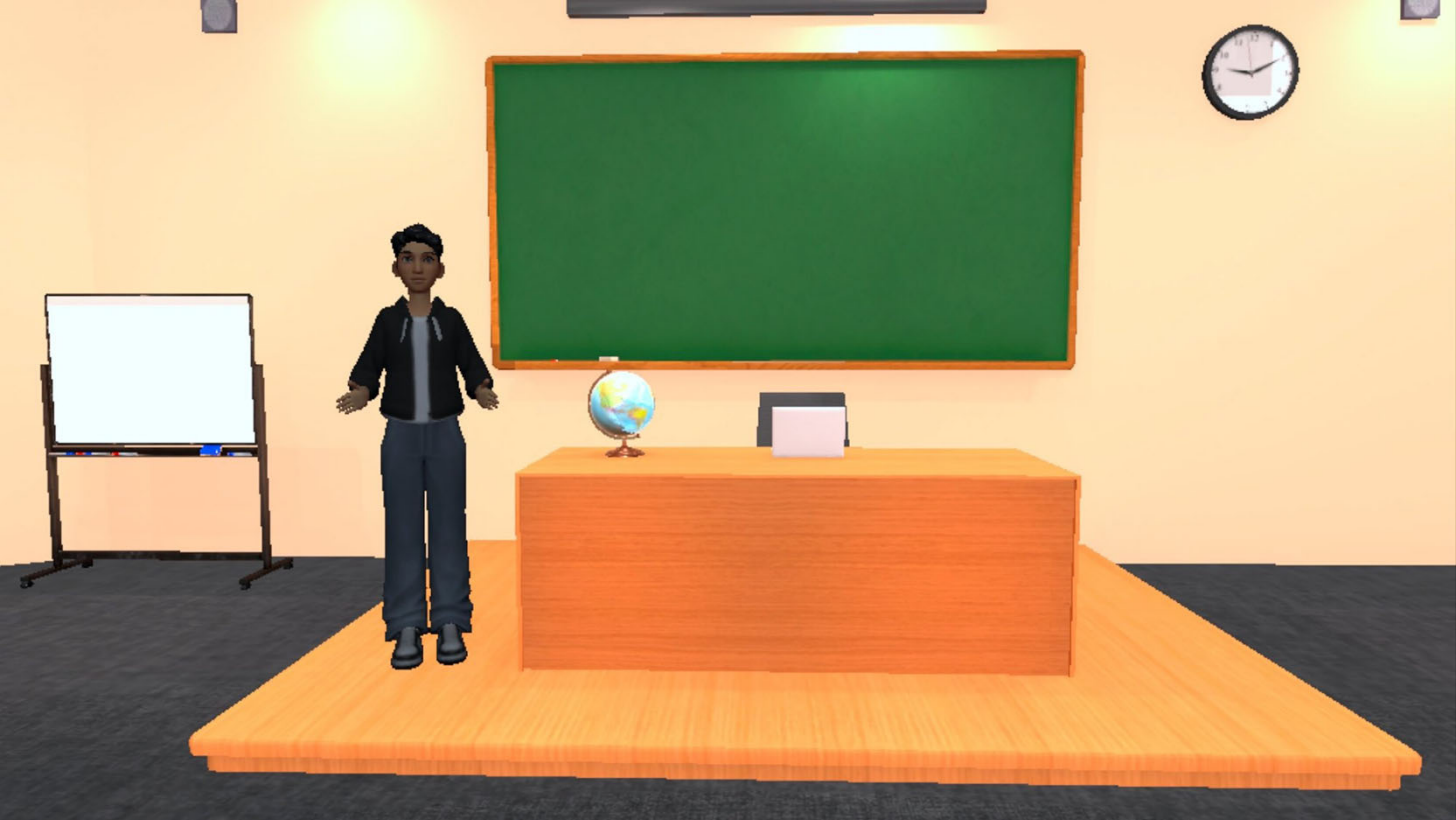
Immersive VR-based language learning system.

Figure 8 illustrates the immersive VR classroom environment where a 3D avatar stands in a virtual classroom equipped with multilingual AI tools. Users interact with the system by selecting their preferred language through an interactive questionnaire presented within the VR interface; in response, the avatar provides real-time translations in both spoken language and IS-based sign translations of selected words and phrases. The avatar’s animation system supports lip-sync with generated speech output and gestural animation for IS signs, with blending between idle, speaking, and signing states managed through Unity’s Animator Controller.

This environment demonstrates how speech-to-text, text-to-sign translation, and text-to-speech services converge to create equitable, self-directed learning opportunities for both deaf and hearing users. The AI-powered avatar delivers educational content in a simulated classroom environment, translating spoken language into International Sign (IS) within a multilingual VR setting. The environment is designed for equitable access and cross-linguistic interaction, with all AI services hosted on scalable AWS Cloud infrastructure.

**Figure 9.**
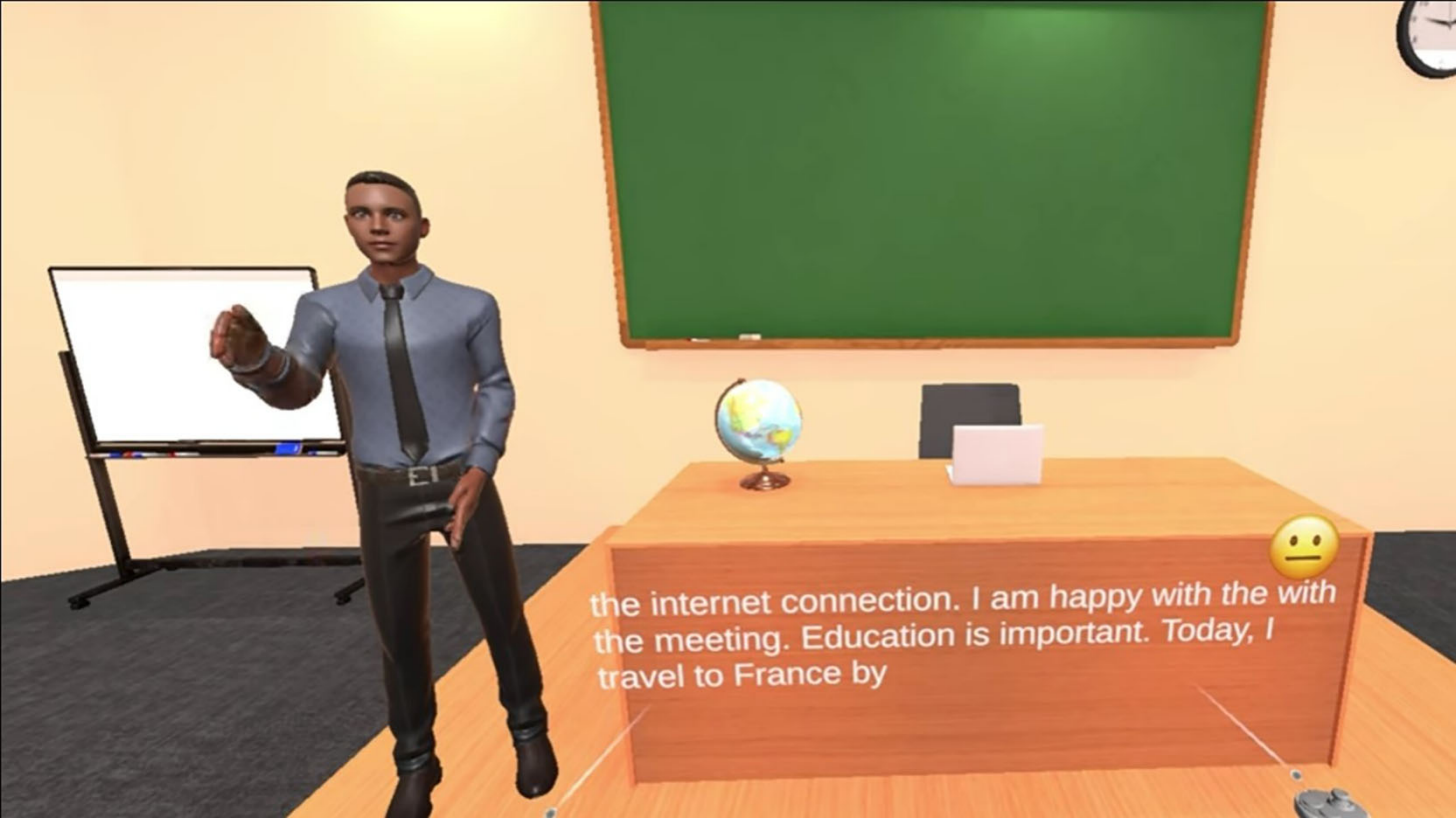
MVP demonstration showing multilingual avatar interaction in VR.

Figure 9 shows a snapshot of the pipeline from voice input to sign and spoken output in the Meta Quest 3 environment. The avatar is shown delivering IS signs in the virtual classroom environment, with text display panels visible for transcription and translation output.

### Companion Pilot Evaluation

The educational scenario presented in this paper was evaluated in a companion pilot study of the INTERACT XR platform, ^50^ which shares the same speech-to-text (Whisper), translation (NLLB), pose-estimation (MediaPipe), sentiment (RoBERTa) and avatar-rendering components. INTERACT was developed in parallel under the CORTEX2 Horizon Europe programme (Grant Agreement No. 101070192) and validated under the ethical oversight of the DASKALOS-APPS Ethics Review Board (DASK-CORTEX2-ERB-2025-003, 14 March 2025), with written informed consent from all participants and consent procedures for deaf participants delivered in International Sign by a qualified interpreter. The pilot engaged ten participants across two sessions: a first session (May 2025; N = 6) with technical experts in AI, HCI and accessibility research, and a second session (June 2025; N = 4) with deaf-community stakeholders, including an ISL educational-material developer, a sign-language interpreter, a business professional and a general user. Prior VR experience varied across the cohort (none: 4; once or twice: 4; moderate: 2). Headline findings are summarised in Table 6.

**Table 6.** Headline outcomes from the companion pilot study of the related INTERACT platform (N = 10) ^50^.

Dimension	Reported value	Notes
Overall user satisfaction	≥92%	Pilot KPI exceeded the ≥85% target ^50^
Mean overall experience	4.6/5.0 (SD 0.52)	Session 1 uniformly 5/5; Session 2 uniformly 4/5 ^50^
Engagement increase ≥51%	8/10	Self-report relative to conventional meetings ^50^
Sentiment-analysis accuracy (perceived ≥86%)	8/10	Subjective rating; 2/10 rated <80% ^50^
Demand for additional languages/sign languages	10/10	Unanimous across both sessions ^50^
Willingness to participate in future testing	9/10	One participant responded "Maybe" ^50^
Most frequent improvement request	Avatar facial expressions (6/10)	Aligns with non-manual-marker limitation in §5 ^50^

## Results

### System Implementation and Integration

The proposed platform successfully integrates six AI-driven services into a cohesive XR learning experience. Table 2 summarises the implemented services, their underlying models, and key performance characteristics.

**Table 2.** Summary of AI Services implemented in the proposed platform.

Service	Model/Technology	Key characteristic
Speech-to-text	OpenAI Whisper ^23^	Multilingual ASR, 680 k + hours training data
Text-to-Text translation	Meta NLLB-200 ^24^	200 languages, Mixture of Experts architecture
Text-to-Speech	AWS Polly	34 languages, Mixture of Experts architecture
Sentiment	RoBERTa	Multi-class emotion classification
Session Summarisation	flan-t5-base-samsum ^43^	Abstractive dialogue summarisation
IS Translation	MediPipe and AVATAR animation ^31^	750 gesture videos, <300 ms animation latency

### TTS Benchmarking

Beyond the latency analysis presented in Table 1, voice quality metrics were also evaluated. Table 3 presents the Mean Opinion Score (MOS) and Word Error Rate (WER) values reported by service providers for the compared TTS services. Table 4 summarises the service capabilities, which are important considerations for ensuring the platform remains accessible. Based on the benchmarking, AWS Polly Standard was selected for the proposed platform due to its lowest and most consistent first-byte latency (50–100 ms), cost-effectiveness ($4 per million characters), and consistent performance across testing sessions, avoiding the high variability observed with other services.

**Table 3.** Voice quality metrics for text-to-speech services.

Service	Mean Opinion Score (MOS)	Word error rate
AWS Polly Standard	3.5–3.8	4.2
Google Cloud Standard	3.2–3.5	Variable
Microsoft Azure	3.8–4.0	3.0
ElevenLabs	3.83–4.2	2.83

**Table 4.** Service capabilities of TTS services (standard models).

Service	Languages	Voices	Max length
AWS Polly Standard	34	66	3000
Google Cloud Standard	40+	220+	5000
Microsoft Azure	140+	110+	5000
ElevenLabs	29	5000+	5000

### Benchmarking NLLB Against EuroLLM

To evaluate potential improvements to the translation component, a comprehensive benchmark comparison was conducted between the deployed NLLB-200-distilled-600 M model and two variants of the EuroLLM 1.7B model. All experiments were performed on a consumer-grade workstation equipped with an NVIDIA GeForce RTX 4060 GPU (8 GB VRAM), ensuring that the benchmarking conditions reflect realistic deployment scenarios rather than high-end server infrastructure. The benchmarking used a test dataset of 10 English-to-French translations with varying complexity levels: simple conversational phrases (3 examples), medium-complexity technical sentences (4 examples), and complex sentences with specialised terminology (3 examples). BLEU was computed against a single human-authored reference using SacreBLEU v2.4.0 with the default 13a tokeniser, with each translation repeated three times to assess inference-time variance. The 10-sentence test bed is acknowledged as a small benchmark, and the comparison should be read as an initial side-by-side rather than a definitive evaluation; a planned extension to FLORES-200 and EuroParl-style domains (EN–FR, EN–EL, EN–ES, EN–DE) is the subject of a dedicated follow-up study. Models were loaded sequentially to manage the limited GPU memory, and inference was conducted using float16 precision to maximise throughput within the available VRAM. For each model, translation quality (BLEU scores), inference speed, and resource utilisation were measured. Table 5 presents the comparative results.

**Table 5.** NLLB vs EuroLLM performance comparison.

Metric	NLLB-200	EuroLLM 1.7B Base	EuroLLM 1.7B Instruct
Average BLEU Score	79.25	27.58	84.34
Average Translation Time (s)	0,596	1.509	0.529
Model Load Time (s)	26.63	25.37	40.99
Memory Usage (GB)	~2.5	~3.5	~3.5
Successful Translations	10/10	10/10	10/10

The key findings from the benchmarking are as follows. The EuroLLM 1.7B Instruct model achieved the highest BLEU score (84.34), outperforming NLLB (79.25) by approximately 5 points, demonstrating superior translation quality for European language pairs. However, the EuroLLM Base model performed poorly (27.58), highlighting the critical importance of instruction tuning for translation tasks. In terms of inference speed, EuroLLM 1.7B Instruct demonstrated the fastest average translation time (0.529 s), marginally faster than NLLB (0.596 s), while the base model was significantly slower at 1.509 s per translation. Purpose-built sequence-to-sequence models (NLLB) showed strong performance, while causal LM models averaged 61.37 BLEU across all variants. The EuroLLM models require more memory (~3.5 GB vs. ~2.5 GB for NLLB’s distilled version), with longer initial loading times for the instruction-tuned variant. The results present a compelling case for considering EuroLLM 1.7B Instruct as an alternative to NLLB in the translation pipeline, particularly given its specialised focus on European languages that aligns with the proposed platform’s target markets.

### End-to-End Pipeline Latency

To characterise end-to-end performance, the full processing chain was instrumented and wall-clock latency was measured across two scenarios: (A) speech-in → translated-speech-out (hearing pathway: Whisper → NLLB → AWS Polly → audio playback) and (B) speech-in → IS-signed-out (deaf pathway: Whisper → NLLB → IS lookup/classifier → avatar animation). Measurements were collected on a clean LAN connection to AWS eu-west-1 over 200 utterances per scenario (mean utterance length 17.4 words; English source, French target), with per-stage timestamps logged at the entry and exit of each microservice so that total wall-clock latency is inclusive of network round-trips. Scenario A yielded a total latency of 2.05 ± 0.31 s (Whisper 1.18 ± 0.21 s; NLLB 0.60 ± 0.09 s; Polly 0.25 ± 0.06 s; Unity playback 0.02 ± 0.01 s) and scenario B 2.32 ± 0.34 s (Whisper 1.18 ± 0.21 s; NLLB 0.60 ± 0.09 s; IS lookup and animation 0.28 ± 0.07 s; Unity rendering 0.02 ± 0.01 s). Both totals fall within commonly accepted thresholds for conversational educational use and are consistent with the sub-300 ms IS animation budget reported above and with per-component latencies independently observed. ^50^ Whisper transcription is the dominant cost in both pathways, indicating that further work on speech-recognition acceleration would yield the largest user-perceived gains.

### Cross-Referenced Pilot Findings

Headline outcomes from the companion pilot are summarised in Table 6. Across the ten participants, 90% rated their overall experience at 4/5 or 5/5 (combined mean 4.6/5.0, SD 0.52), 80% reported an engagement increase of at least 51% relative to conventional meetings, and 100% requested expanded language and sign-language support, directly aligning with the multilingual-IS focus of the platform described here. Qualitative themes most frequently raised were avatar facial-expression integration (6/10), adjustable signing speed and replay (4/10), larger avatar sizing and improved subtitle contrast (3/10), and avatar customisation (2/10). Because the AI service layer is shared between the two studies, these findings are directly informative about the perceived usability and accessibility value of the AI services described here; the N = 10 cohort is small, and the reported values are descriptive of the participating sample rather than indicative of population-level effectiveness.

## Discussion

The results demonstrate that the proposed platform successfully integrates multiple AI-driven services into a cohesive XR learning environment that addresses both the communication needs of hearing users and the accessibility requirements of deaf users. The modular, service-oriented architecture enables independent scaling and updating of individual components, which is critical for maintaining system performance as user demands grow and AI models evolve.

The successful integration of six AI services, speech-to-text, text-to-text translation, text-to-speech, sentiment analysis, session summarisation, and IS translation, within a unified XR environment validates the premise that combining multiple AI modalities within immersive settings can create meaningful, accessible learning experiences. The IS-focused approach, leveraging International Sign Language as a lingua franca rather than a single national sign language, has the potential to broaden accessibility beyond national sign language boundaries and serve deaf learners from diverse linguistic backgrounds.

The avatar-mediated sign language delivery, while achieving animation latency under 300 milliseconds, represents an initial proof of concept that would benefit from further refinement. In particular, the quality of non-manual markers (facial expressions, head movements, body posture) is recognised in the broader literature as essential for comprehensible and natural signing, ^16, 19^ and their integration into the avatar system represents an important area for future development. This same limitation emerged as the highest-priority enhancement in the companion pilot feedback. ^50^

The benchmarking analyses provide quantitative evidence for technology selection decisions. The TTS evaluation confirmed that AWS Polly offers the best balance of latency, cost, and consistency for real-time VR applications, while the NLLB vs. EuroLLM comparison revealed that instruction-tuned causal language models can outperform purpose-built translation models in both quality and speed for European language pairs. This finding has implications not only for the proposed platform but also for the broader UTTER project’s development of European language models. The end-to-end latency analysis further confirms that the full pipeline, including all microservice and network costs, fits within typical conversational thresholds for both user pathways, with speech recognition identified as the dominant latency cost.

Several limitations should be acknowledged. The IS dataset of 750 gesture videos, while sufficient for an initial proof-of-concept, represents a limited vocabulary that constrains the expressiveness of the translation system, and the current dataset split does not enforce an inter-signer hold-out; both will be addressed in the planned dataset expansion. The translation benchmark in Table 5 is based on only ten EN–FR sentences, so the 5-point BLEU advantage of EuroLLM 1.7B Instruct over NLLB-200 should be regarded as suggestive only until the planned FLORES-200 evaluation is completed. Additionally, formal usability studies with standardised instruments (e.g., System Usability Scale, NASA-TLX for cognitive load) have not yet been conducted on the language-learning configuration described here and represent an important direction for future validation. The system has not yet been evaluated with end users wearing VR headsets in naturalistic, longitudinal learning scenarios, which is necessary to assess immersion, spatial presence, and actual learning outcomes.

The ethical dimensions of the platform were addressed through adherence to GDPR regulations, anonymisation of voice and gesture data, secure API access controls, and the application of FAIR data principles. Efforts were made to ensure fairness across languages and cultural contexts, particularly in the design and training of sign language avatars, to avoid misrepresentation and bias.

The platform presented in this study addresses a key gap identified in the literature: the absence of integrated, multimodal AI systems within XR that serve both deaf and hearing learners simultaneously. While previous work has typically focused on individual AI capabilities in isolation, such as speech recognition for captioning ^23^ or sign language recognition for classification, ^16, 46^ this system demonstrates the feasibility of orchestrating multiple AI services into a unified educational experience. This approach aligns with the vision articulated by Hirzle et al., ^21^ who identified the convergence of XR and AI as a high-potential research frontier, and extends it by grounding the integration in a concrete, deployable platform validated through comprehensive technical. benchmarking and complementary pilot evidence. ^50^

From a policy perspective, the platform’s focus on IS as a lingua franca for deaf communication across national boundaries aligns with the objectives of the European Accessibility Act and the EU’s commitment to digital inclusion. By providing an XR-based learning environment that supports both spoken multilingual interaction and sign language translation, the proposed platform contributes to the broader agenda of equitable access to education, as articulated in the EU Digital Education Action Plan (2021–2027). The modular, cloud-native architecture ensures that the platform can be adapted to diverse institutional contexts, from formal language schools to informal community-based learning settings. Furthermore, the same underlying AI services have demonstrated applicability beyond language education, including accessible communication in professional and business meeting contexts. ^47^ and inclusive teleconferencing. ^50^

## Conclusions

This paper presented a comprehensive framework for integrating modular AI-driven services into immersive XR environments to support language learning for both deaf and hearing individuals. The platform combines speech-to-text transcription, multilingual translation, text-to-speech synthesis, sentiment analysis, and International Sign translation, all delivered through AI-powered 3D avatars within a Unity-based VR environment deployed on Meta Quest 3 headsets.

The system’s modular architecture and successful integration of all AI components demonstrate the technical feasibility of the approach. Quantitative benchmarking of TTS services and multilingual translation models, together with the end-to-end pipeline latency analysis (2.05 s hearing pathway; 2.32 s IS pathway) and complementary pilot-validation evidence from the related INTERACT deployment, ^50^ provided evidence-based justification for technology selection and identified promising alternatives for future integration.

Future work will focus on several priorities. First, the IS vocabulary will be significantly expanded by collecting and processing additional gesture videos from diverse interpreters, with improved gesture landmark normalisation to ensure smoother and more consistent avatar animations across signers, and the gesture dataset will be re-split with strict inter-signer hold-out to enable rigorous generalisation testing. Second, avatar facial expressions and lip-sync functionality will be developed to provide the non-manual markers that are essential for natural sign language communication. Third, the single-user experience will be extended into a multiplayer VR setting where hearing and deaf users can communicate in real time, better showcasing the integrated sentiment analysis and translation capabilities and enabling collaborative language learning scenarios. Fourth, formal user studies with larger and more diverse participant groups will be designed and conducted, incorporating standardised usability instruments (e.g., System Usability Scale, NASA-TLX), pre/post learning assessments, and cognitive load measurements to produce quantitative evidence of learning effectiveness, with sample size determined by a priori power analysis. Fifth, the EuroLLM 1.7B Instruct model will be further evaluated for integration into the production translation pipeline, with expanded benchmarking across additional European language pairs (at minimum the FLORES-200 EN–FR/EL/ES/DE sets) and domain-specific terminology. Sixth, the platform architecture will be extended to support additional XR hardware beyond Meta Quest 3, including standalone AR devices and desktop-based VR systems, to maximise accessibility across institutional contexts. Finally, the platform will be piloted in formal educational settings, including schools and language training centres, to assess real-world adoption, learning outcomes, and alignment with the EU Digital Education Action Plan’s goals for inclusive, technology-enhanced learning.

## Declaration of Generative AI and AI-assisted Technologies in the Writing Process

During the preparation of this manuscript, the authors used generative AI-based tools, specifically large language models such as ChatGPT (OpenAI), solely to assist with language editing and consistency checks in limited sections. All substantive decisions regarding the design of the review, screening, data extraction, analysis and interpretation were made by the authors. The authors carefully reviewed and edited all AI-assisted text and take full responsibility for the integrity and accuracy of the manuscript’s content.

## Open Access Software and AI Models

All core AI models implementations central to the novelty and findings of this research utilise open-source libraries available under permissive licences for reuse:

• **Speech Recognition (OpenAI Whisper):** https://github.com/openai/whisper (MIT License)
• **Text to Text Translation (Meta AI NLLB):** https://github.com/facebookresearch/fairseq/tree/nllb (MIT License)
• **Pose Estimation (Google MediaPipe):** https://github.com/google/mediapipe (Apache License 2.0)
• **Sentiment Analysis:** Hugging Face Transformers— https://huggingface.co/j-hartmann/emotion-english-distilroberta-base (Apache License 2.0)
• **Summarization:** Hugging Face BART— https://huggingface.co/philschmid/bart-large-cnn-samsum (MIT License)
• **Multilingual Translation Benchmarking (EuroLLM-1.7B Base):** https://huggingface.co/utter-project/EuroLLM-1.7B (Apache License 2.0)
• **Multilingual Translation Benchmarking (EuroLLM-1.7B-Instruct):** https://huggingface.co/utter-project/EuroLLM-1.7B-Instruct (Apache License 2.0)

These repositories contain the complete source code, model configurations, and training procedures necessary to replicate the AI integration methodology described in this paper.

## Ethics and Consent Statement

This research did not involve direct experimentation with human participants. The International Sign (IS) gesture dataset used in this study was curated from publicly available, open-source video resources, specifically HandSpeak and SpreadTheSign, which are freely accessible online for research and academic purposes under permissive licences. No new video recordings of human signers were produced as part of this work. The gesture videos were processed computationally using Google MediaPipe to extract anonymised hand landmark coordinates; no personally identifiable information was retained in the resulting dataset. The AI models employed in the platform (Whisper, NLLB, RoBERTa, flan-t5-base-samsum) are publicly available open-source models used in their original or fine-tuned forms, and no additional training on personal data was conducted. The system architecture and all API services were designed with privacy-by-design principles, processing user inputs transiently without storing personally identifiable speech, text, or gesture data, in compliance with the European Union General Data Protection Regulation (GDPR). No adverse events were reported during the development and testing of the platform. As no human participants were involved in any stage of this research, ethical approval and informed consent were not required.

## Data Availability

No data are associated with this article. This study did not generate or analyse datasets involving human participants. The platform’s functionality was validated through technical benchmarking using publicly available AI models and synthetic test datasets as described in the Methods section. The IS gesture dataset developed for avatar animation is released as an open resource to support further research in sign language translation and accessibility technology development. The complete dataset and code necessary to replicate the IS animations are available in Zenodo: [ 10.5281/zenodo.18656296]. ^49^ This dataset is released under a CC-BY 4.0 licence. Data are available under the terms of the Creative Commons Attribution 4.0 International licence (CC-BY 4.0).
