# Supplementary material for: AI-based services for inclusive language learning in immersive XR environments: Speech translation, and sign language integration
Source: Open Res Eur. 2026 Jun 13;6:72. Originally published 2026 Mar 11. [Version 2] doi: 10.12688/openreseurope.23214.2 (PMC13324358; doi:10.12688/openreseurope.23214.2)
Supplement: Peer review history

# Reviewer report: Reviewer response for version 2

**DOI:** 10.21956/openreseurope.26092.r75959

**Published:** 2026-07-01

**Authors:** Daniel Cabeza-Campillo

**Affiliations:** 1 University of Warwick, Coventry, England, UK

The article entitled “AI-based services for inclusive language learning in immersive XR environments: Speech translation, and sign language integration” examines the usefulness of Extended Reality (XR) in language education and investigates the integration of AI-based services to foster inclusive language learning in this context, addressing the needs of deaf and hard-of-hearing individuals. More specifically, as Table 2 (p. 14) illustrates, the study implements six AI services within a proposed platform: OpenAI Whisper, Meta NLLB-200, AWS Polly, RoBERTa, flan-t5-base-samsum and MediPipe and AVATAR animation. The way these AI services are implemented is clearly illustrated in Figure 1 (p. 7).

 The introduction of the study reviews some of the main historical pedagogical approaches to language learning, including the grammar-translation approach, the audio-lingual methods and the immersion-based strategies. The introduction also acknowledges that, nowadays, the use of technological tools in language learning allows learners to practise independently, which may improve their speaking confidence and reduce anxiety by offering “risk-free, repeatable practice opportunities” (p. 4).

 However, the introduction also recognizes that, “while AI-driven avatars hold great promise for natural, culturally sensitive language instruction, they currently struggle to deliver accurate interactions across different languages and cultural nuances due to challenges in NLP algorithms and the complexity of implementing diverse linguistic databases” (p. 4).

 This study addresses a much needed evaluation of the implementation of AI-based services for inclusive language learning in immersive XR environments to address the needs of deaf and hard-of-hearing individuals. The data has been analysed in a precise way and the discussion of results clearly answers the research questions.

Is the study design appropriate and does the work have academic merit?

Yes

Is the work clearly and accurately presented and does it cite the current literature?

Yes

If applicable, is the statistical analysis and its interpretation appropriate?

Yes

Are all the source data underlying the results available to ensure full reproducibility?

Yes

Are the conclusions drawn adequately supported by the results?

Yes

Are sufficient details of methods and analysis provided to allow replication by others?

Yes

Reviewer Expertise:

Translation Studies; News Translation; Metaphor Studies; Critical Discourse Analysis; Spanish Language.

I confirm that I have read this submission and believe that I have an appropriate level of expertise to confirm that it is of an acceptable scientific standard.

# Reviewer report: Reviewer response for version 2

**DOI:** 10.21956/openreseurope.26092.r75841

**Published:** 2026-06-25

**Authors:** Diego Resende Faria

**Affiliations:** 1 Computer Science, Loughborough University Department of Computer Science (Ringgold ID: 152591), Loughborough, England, UK

The revised manuscript is **substantially stronger than the original version** and is now much more appropriately positioned as a:

**System demonstration and technical feasibility study** rather than a validation of educational effectiveness.

 This was one of the main concerns in the original review, and the authors explicitly state this repositioning in the "Amendments from Version 1" section and throughout the paper.

 Compared with the original submission, the revised manuscript demonstrates substantial improvements in reproducibility, methodological transparency, technical validation, and scientific positioning. Key additions include a detailed dataset and preprocessing description, explicit model training procedures, end-to-end latency evaluation, expanded benchmarking methodology, and a more balanced discussion of limitations.

 Importantly, the work is now appropriately framed as a technical feasibility study rather than an educational effectiveness evaluation. The revised version is considerably stronger and addresses the majority of the concerns raised during peer review.

Is the study design appropriate and does the work have academic merit?

Partly

Is the work clearly and accurately presented and does it cite the current literature?

Partly

If applicable, is the statistical analysis and its interpretation appropriate?

Partly

Are all the source data underlying the results available to ensure full reproducibility?

Partly

Are the conclusions drawn adequately supported by the results?

Partly

Are sufficient details of methods and analysis provided to allow replication by others?

Partly

Reviewer Expertise:

Human-Robot Interaction, Health AI, Child-Machine Interaction, Neuro-Affective Intelligence, Bayesian Machine Learning, Cognitive Robotics, Emotion AI, Affective Computing, Human Activity Recognition.

I confirm that I have read this submission and believe that I have an appropriate level of expertise to confirm that it is of an acceptable scientific standard.

# Reviewer report: Reviewer response for version 1

**DOI:** 10.21956/openreseurope.25118.r71511

**Published:** 2026-04-28

**Authors:** Diego Resende Faria

**Affiliations:** 1 Computer Science, Loughborough University Department of Computer Science (Ringgold ID: 152591), Loughborough, England, UK

This paper presents a technically well-engineered and timely contribution at the intersection of **XR, multimodal AI, and accessibility**. The integration of speech, translation, sentiment, and sign language into a unified immersive environment is novel and relevant, particularly in the context of inclusive education.

 However, the work currently remains **primarily an engineering prototype with limited scientific validation**. The absence of user studies, limited statistical rigor, and insufficient evaluation of the sign language component significantly weaken the paper from a research perspective.

 The manuscript would benefit from a clearer positioning as a **system demonstration and technical feasibility study**, unless additional experimental validation is provided.

**Strengths**

- Strong multimodal integration (rare in XR literature)
- Clear system architecture and implementation
- Relevant accessibility contribution (IS focus)
- Good engineering validation (latency, scalability)
- Alignment with EU policy and societal impact

**Weaknesses**

- Lack of user-centered evaluation
- Weak statistical validation
- Limited dataset size and benchmarking rigor
- Missing technical details for reproducibility
- Overstated claims about learning effectiveness

**Specific Suggestions for Improvement**

**Must Address (Critical)**

1. Add quantitative evaluation of IS translation model
2. Expand benchmarking datasets (translation, TTS)
3. Include statistical analysis with proper metrics
4. Clarify reproducibility:
  - Model architectures
  - Training details
5. Reframe conclusions to match evidence

**Recommended**

1. Conduct user studies (even small-scale pilot)
2. Add end-to-end system evaluation
3. Include comparison with baseline systems
4. Improve discussion on:
  - Limitations of IS dataset (750 samples is very small)
  - Generalization issues

**improvements:**

- Provide:
  - Full ML training pipeline (architecture, loss, optimizer, epochs)
  - Dataset split (train/val/test)
  - Evaluation metrics for IS translation
- Expand benchmarking dataset significantly
- Provide end-to-end latency analysis

The paper has **strong potential**, but requires **substantial improvement in scientific validation, statistical rigor, and reproducibility** to meet the standards expected for Open Research Europe.

Is the study design appropriate and does the work have academic merit?

Partly

Is the work clearly and accurately presented and does it cite the current literature?

Partly

If applicable, is the statistical analysis and its interpretation appropriate?

Partly

Are all the source data underlying the results available to ensure full reproducibility?

Partly

Are the conclusions drawn adequately supported by the results?

Partly

Are sufficient details of methods and analysis provided to allow replication by others?

Partly

Reviewer Expertise:

Human-Robot Interaction, Health AI, Child-Machine Interaction, Neuro-Affective Intelligence, Bayesian Machine Learning, Cognitive Robotics, Emotion AI, Affective Computing, Human Activity Recognition.

I confirm that I have read this submission and believe that I have an appropriate level of expertise to state that I do not consider it to be of an acceptable scientific standard, for reasons outlined above.
